# Potential differences in ephedrine requirements between left lateral and right lateral decubitus positions during neuraxial anesthesia for cesarean delivery

**DOI:** 10.3389/fmed.2024.1454681

**Published:** 2024-10-10

**Authors:** Shanshan Wu

**Affiliations:** Clinical Laboratory Department, The Affiliated Hospital of Liaoning University of Traditional Chinese Medicine, Shenyang, Liaoning, China

**Keywords:** cesarean delivery, neuraxial anesthesia, hemodynamics, vasopressor, lateral decubitus positioning

## Abstract

The recent article by Wen et al., published in PLOS ONE, titled “*Effects of neuraxial anesthesia in sitting and lateral positions on maternal hemodynamics in cesarean section: A systematic review and meta-analysis*,” caught my attention. In their study, the authors observed the effects of neuraxial anesthesia in sitting and lateral positions on maternal hemodynamics during cesarean section. Given the anatomical differences between the left and right sides of the body, which could result in differences in maternal hemodynamics and vasopressor requirements during neuraxial anesthesia for cesarean delivery, I was intrigued by the idea of further dividing the lateral position data from Wen et al.'s study into three subgroups: “left lateral position,” “right lateral position,” and “not mentioned” (where the included original study did not mention the lateral position) for a subgroup analysis. It seems to be more rigorous, the subgroup analysis revealed that the usage rate of ephedrine support was 1.42 times higher for parturients in the right lateral position compared to those in the sitting position. This finding supports our recommendation to distinguish between left and right lateral decubitus positioning in neuraxial anesthesia for cesarean delivery. But in contrast, no significant difference was observed between the sitting and lateral positions in terms of the number of parturients requiring ephedrine in Wen et al.'s. Given the limited research on the right-lateral position and its hemodynamic effects, further studies are needed to explore its clinical applications. Future research should also focus on conducting larger trials with greater sample sizes to evaluate the long-term neonatal outcomes associated with varying maternal positions. Additionally, researchers should conduct subgroup analyses that separate the left- and right-lateral positions to provide clearer guidance for anesthesiologists.

## 1 Introduction

Cesarean section is a common mode of delivery, and the choice of anesthesia plays a critical role in ensuring the safety and comfort of both mother and baby. Neuraxial anesthesia is widely used in cesarean sections due to its effective analgesic properties and maternal-fetal safety (2). Patient positioning is an important factor in anesthesia management, as it can significantly affect the anesthesia's effectiveness and the patient's hemodynamic status (3). Neuraxial anesthesia can be administered in a sitting, lateral, or rarely prone posture. Each position has its advantages and disadvantages.

The advantage of the sitting position is that midline structures can be easily identified, even in obese patients, and it prevents compression of the aorta and the inferior vena cava, thereby avoiding supine hypotension syndrome caused by sympathetic blockade. However, maintaining the sitting position is difficult in cases of multiple pregnancies, umbilical cord prolapse, and emergency situations, where the lateral position may be more suitable for the patient. Sympathectomy caused by neuraxial anesthesia, combined with peripheral blood pooling due to gravity, makes hypotension more pronounced in the sitting position than in the lateral position (4). The lateral position, which has a lower risk of postural hypotension, is better suited for patients in a weakened condition and provides superior local anesthetic spread, leading to better sensory blockade. However, the lateral position may cause compression of the axillary artery, axillary vein, and brachial plexus in the armpit area (5).

Cesarean section under spinal anesthesia often results in maternal hypotension and fetal acidosis. The hypotension experienced by mothers after neuraxial anesthesia is mainly due to the spread of local anesthetics in the subarachnoid space and compression of the abdominal aorta by the uterus (6). Although current practice suggests a 15° left-lateral tilt for cesarean sections under neuraxial anesthesia, some early studies reported the use of a right-lateral tilt in actual patients. The effects of left-lateral and right-lateral tilt on the aortic and inferior vena cava volumes in pregnant women remain unclear. To address this, Fujita et al. used magnetic resonance imaging (MRI) and found no significant difference in aortic volume between the left-lateral and right-lateral tilt positions under neuraxial anesthesia. However, the mean inferior vena cava volume in the 30° left-lateral tilt was significantly greater than that in the 15° and 30° right-lateral tilt positions. Interestingly, for some patients, the 30° right-lateral tilt yielded the optimal inferior vena cava volume (7).

These findings suggest that the hemodynamic effects of left-lateral or right-lateral tilt following neuraxial anesthesia differ among individuals and are directly related to the selection and dosage of vasopressors used in treatment.

## 2 Statistical methods

First, we verified that the extracted data from Wen et al. (1) matched the original data in the included studies. All data were analyzed using RevMan version 5.3 software. Continuous variables such as the lowest systolic blood pressure and ephedrine dose were expressed as mean difference (MD) with a 95% confidence interval (CI). Relative risk (RR) with 95% CI was used for dichotomous variables such as the incidence of hypotension, the usage rate of ephedrine, and the incidence of nausea and vomiting. The I^2^ value indicated the heterogeneity among included original studies; I^2^ values of over 25%, 50%, and 75% are commonly defined as low, medium, and high heterogeneity, respectively. When I^2^ ≥ 50%, the heterogeneity is significant, this degree of variability required sensitivity analysis or subgroup analysis to identify plausible sources of heterogeneity, and the random effect model is applied. When I^2^ <50%, the heterogeneity among included studies is considered small, and the fixed effects model is selected.

## 3 Subgroup analysis results

### 3.1 Incidence of hypotension

Hypotension was observed in the sitting and lateral decubitus groups across seven trials, with the lateral decubitus group divided into right lateral and left lateral decubitus subgroups (8–14). Subgroup analysis showed no difference between the sitting and right lateral decubitus groups (RR, 1.05; 95% CI, 0.26–4.19; *P* = 0.95; I^2^ = 86%), and no difference between the sitting and left lateral decubitus groups (RR, 0.73; 95% CI, 0.54–1.00; *P* = 0.05; I^2^ = 53%) (Supplementary Figure S1).

### 3.2 Lowest systolic blood pressure

Comparing the lowest systolic blood pressure in women after neuraxial anesthesia in the two positions across 5 trials (8–11, 13), subgroup analysis showed no difference between the sitting and right lateral decubitus groups (MD, −2.10; 95% CI, −22.67–18.46; *P* = 0.84; I^2^ = 92%), and no difference between the sitting and left lateral decubitus groups (MD, 1.11; 95% CI, −5.15–7.38; *P* = 0.73; I^2^ = 84%) (Supplementary Figure S2).

### 3.3 Dose of ephedrine

The comparison of ephedrine dosages in nine RCTs after neuraxial anesthesia in parturients in two positions (8–12, 15–18). In Yun et al.'s study (18), it was not mentioned whether the parturients adopted the left or right lateral decubitus position after neuraxial anesthesia, so this study was separately defined as the “not mentioned” subgroup. Subgroup analysis showed that there was no difference between the sitting and right lateral decubitus position groups (MD, 1.73; 95% CI, −3.87–7.33; *P* = 0.55; I^2^ = 78%), and there was no difference between the sitting and left lateral decubitus position groups (MD, −1.00; 95% CI, −5.85–3.84; *P* = 0.68; I^2^ = 78%) (Supplementary Figure S3).

### 3.4 Usage rate of ephedrine

**Figure 1:**
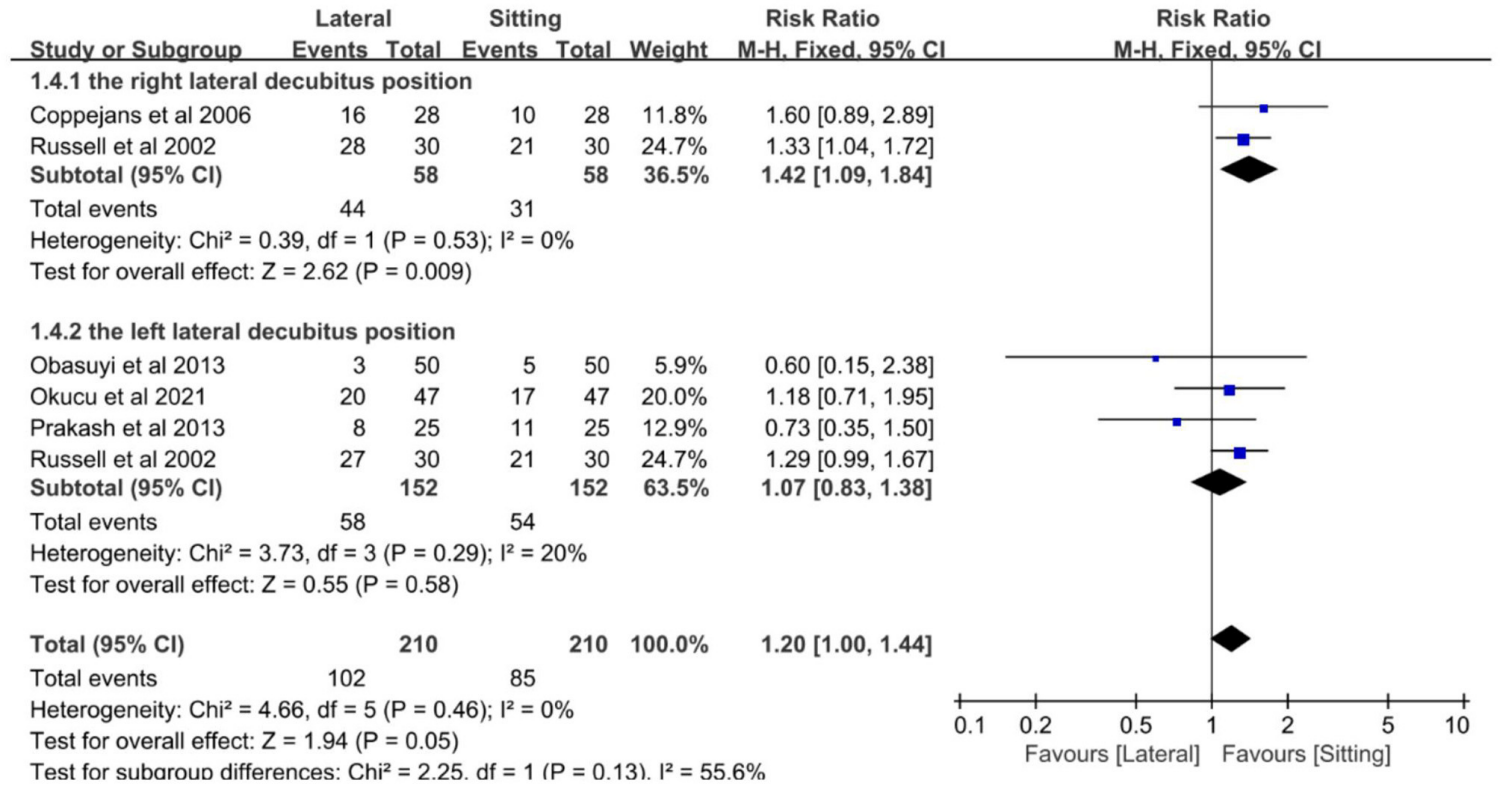
Forest plot of ephedrine requirement.

The number of parturients who needed ephedrine support following spinal anesthesia in the sitting and lateral positions was recorded in five trials (9, 11, 14, 16, 17). Xu et al.'s study (13) was excluded because it did not contain information on the number of parturients using ephedrine after neuraxial anesthesia. Subgroup analysis showed that the likelihood of needing ephedrine support was 1.42 times higher in parturients in the right lateral position compared to those in the sitting position (RR, 1.42; 95% CI, 1.09–1.84; *P* = 0.009; I^2^ = 0%), and there was no difference between the sitting and left lateral position groups (RR, 1.07; 95% CI, 0.83–1.38; *P* = 0.58; I^2^ = 20%) (Figure 1).

### 3.5 Incidence of nausea and vomiting

Comparing the incidence of nausea and vomiting in women after neuraxial anesthesia in the sitting and lateral decubitus positions across nine trials (8, 9, 11–13, 16–19). In Yun et al.'s study (18), it was not mentioned whether the parturients adopted the left or right lateral decubitus position after neuraxial anesthesia, so this study was separately defined as the “not mentioned” subgroup. Subgroup analysis showed no difference between the sitting and right lateral decubitus groups (RR, 1.30; 95% CI, 0.51–3.33; *P* = 0.58; I^2^ = 69%), and no difference between the sitting and left lateral decubitus groups (RR, 0.75; 95% CI, 0.27–2.12; *P* = 0.59; I^2^ = 70%) (Supplementary Figure S4).

## 4 Discussion

The subgroup analysis results showed that the usage rate of women needing ephedrine support was 1.42 times higher in the right lateral decubitus group compared to the sitting group. There was no significant difference in the incidence of hypotension, nausea, and vomiting, lowest systolic blood pressure, ephedrine dose, and probability of needing ephedrine support during cesarean section among the sitting, right and left lateral decubitus groups. The usage rate of ephedrine was higher in the right lateral decubitus group compared to the sitting group, but there was no significant difference between the left lateral decubitus and sitting groups, indicating that the left lateral decubitus position and sitting position can effectively reduce the usage rate of ephedrine than the right lateral decubitus position. However, further large-scale studies are needed to confirm this conclusion due to the limitations of this study, including a small number of included studies and a small sample size.

If hypotension is not corrected promptly with vasopressors, it can lead to nausea, vomiting, decreased uteroplacental blood flow, fetal acidosis, and in rare cases, severe consequences such as cardiovascular collapse. Although ephedrine can effectively treat maternal hypotension, it has higher transplacental transfer than phenylephrine due to its high lipid solubility, leading to fetal sympathetic metabolism stimulation as the dose increases. This results in neonatal acidemia, increased umbilical arterial and venous concentrations of lactate, glucose, epinephrine, and norepinephrine, and increased umbilical venous PCO_2_ (20). A systematic review reported that the risk of fetal acidosis with ephedrine use after neuraxial anesthesia in cesarean section is 5.29 times higher compared to phenylephrine (21). The right lateral decubitus position often requires more vasopressors to maintain blood pressure, increasing the risk of drug-related side effects (22). Given the above situation, studies on the lateral position for cesarean section under neuraxial anesthesia should be divided into the left lateral and the right lateral decubitus subgroups to ensure scientific rigor.

Further research should focus on larger, multicenter RCTs that examine the hemodynamic differences between the left-lateral and right-lateral positions during neuraxial anesthesia. Additionally, studies should explore the long-term outcomes for neonates exposed to different vasopressor regimens, particularly with regard to the risks of neonatal acidosis associated with ephedrine vs. phenylephrine. By advancing our understanding of the relationship between maternal positioning and anesthesia outcomes, we can improve maternal and neonatal safety during cesarean-sections.
