# Supplementary figures and images for: Potential differences in ephedrine requirements between left lateral and right lateral decubitus positions during neuraxial anesthesia for cesarean delivery
Source: Front Med (Lausanne). 2024 Oct 10;11:1454681. doi: 10.3389/fmed.2024.1454681 (PMC11499170; doi:10.3389/fmed.2024.1454681)

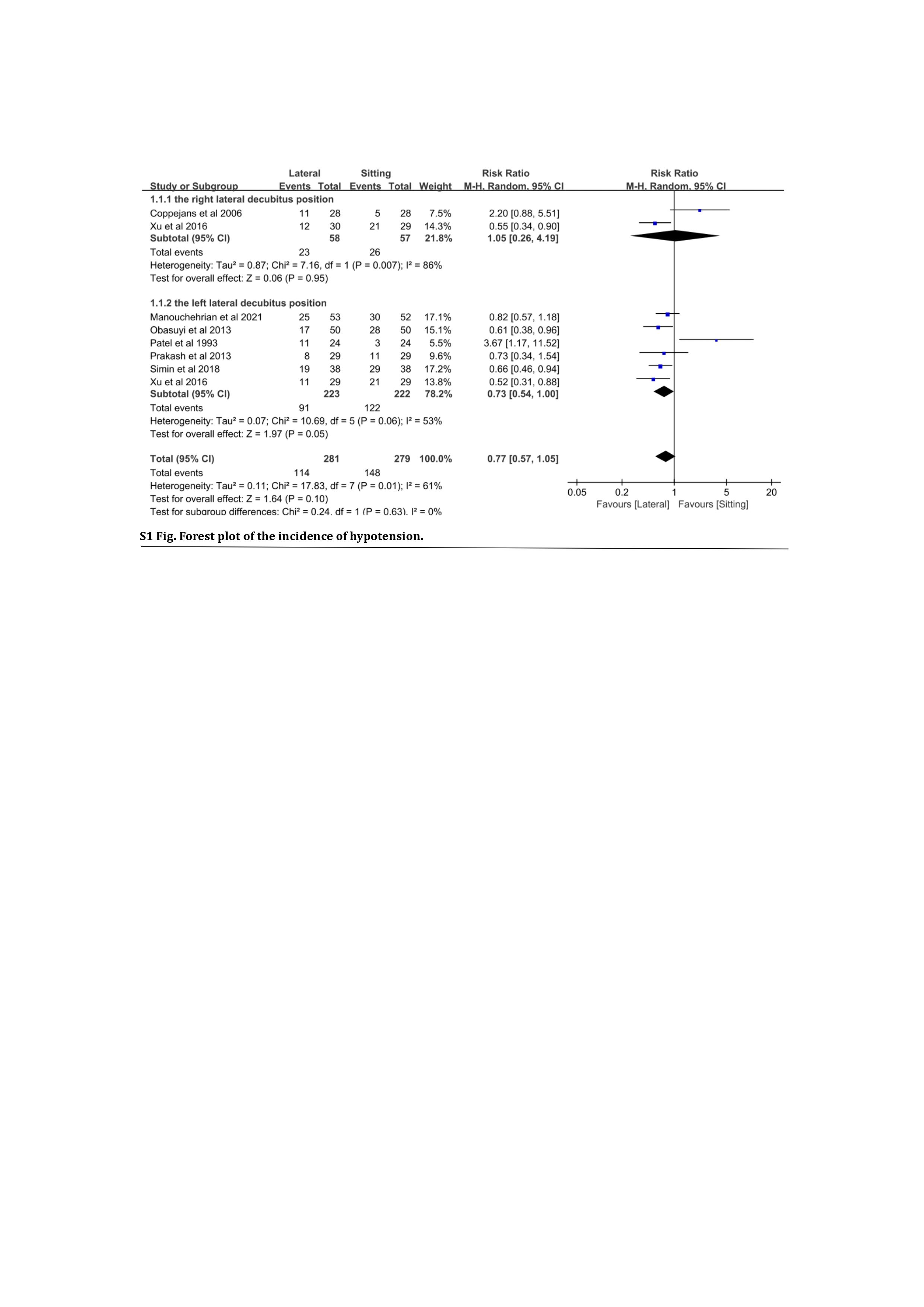

Supplement: Supplementary file 1 [file Image_1.JPEG]

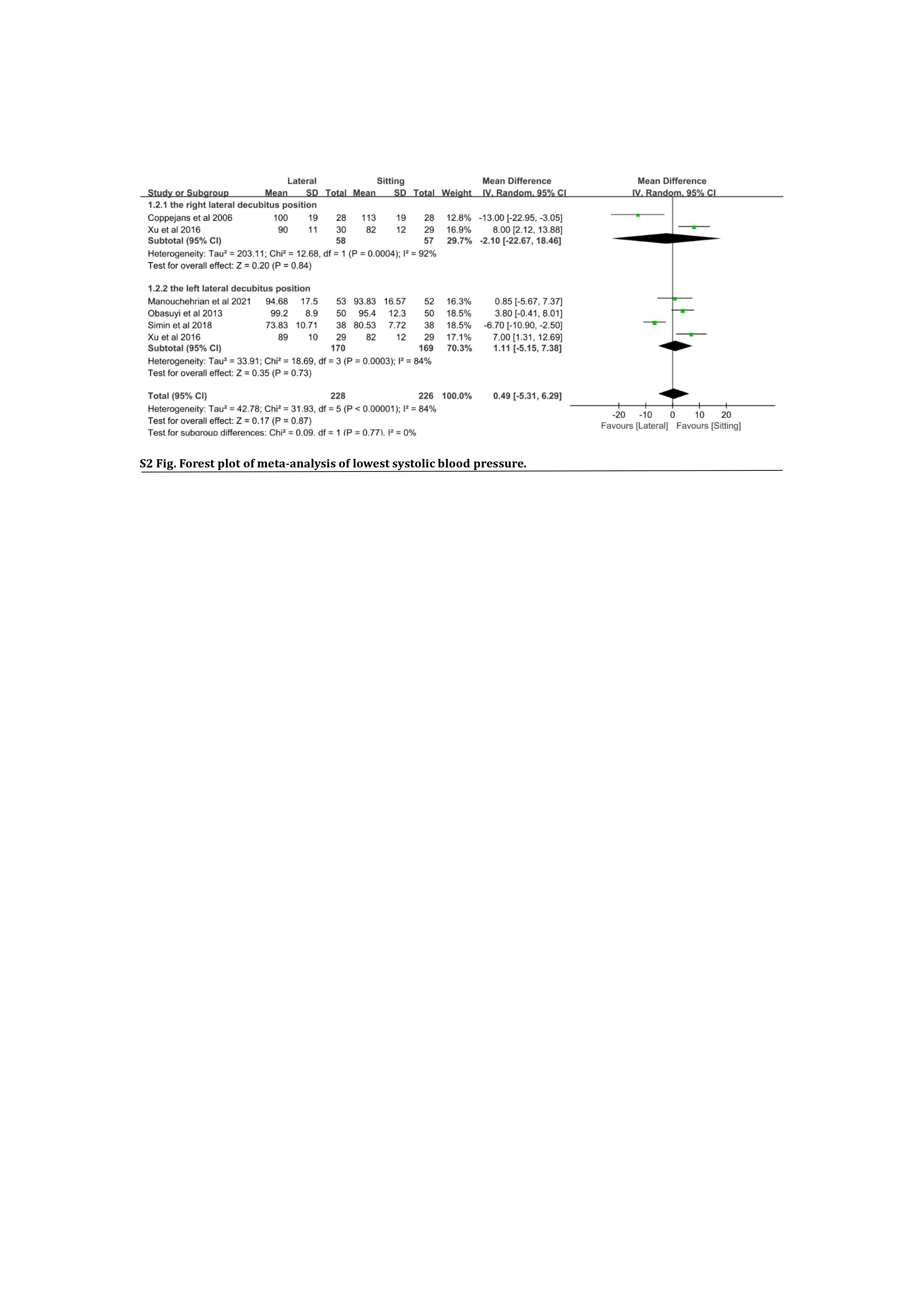

Supplement: Supplementary file 2 [file Image_2.JPEG]

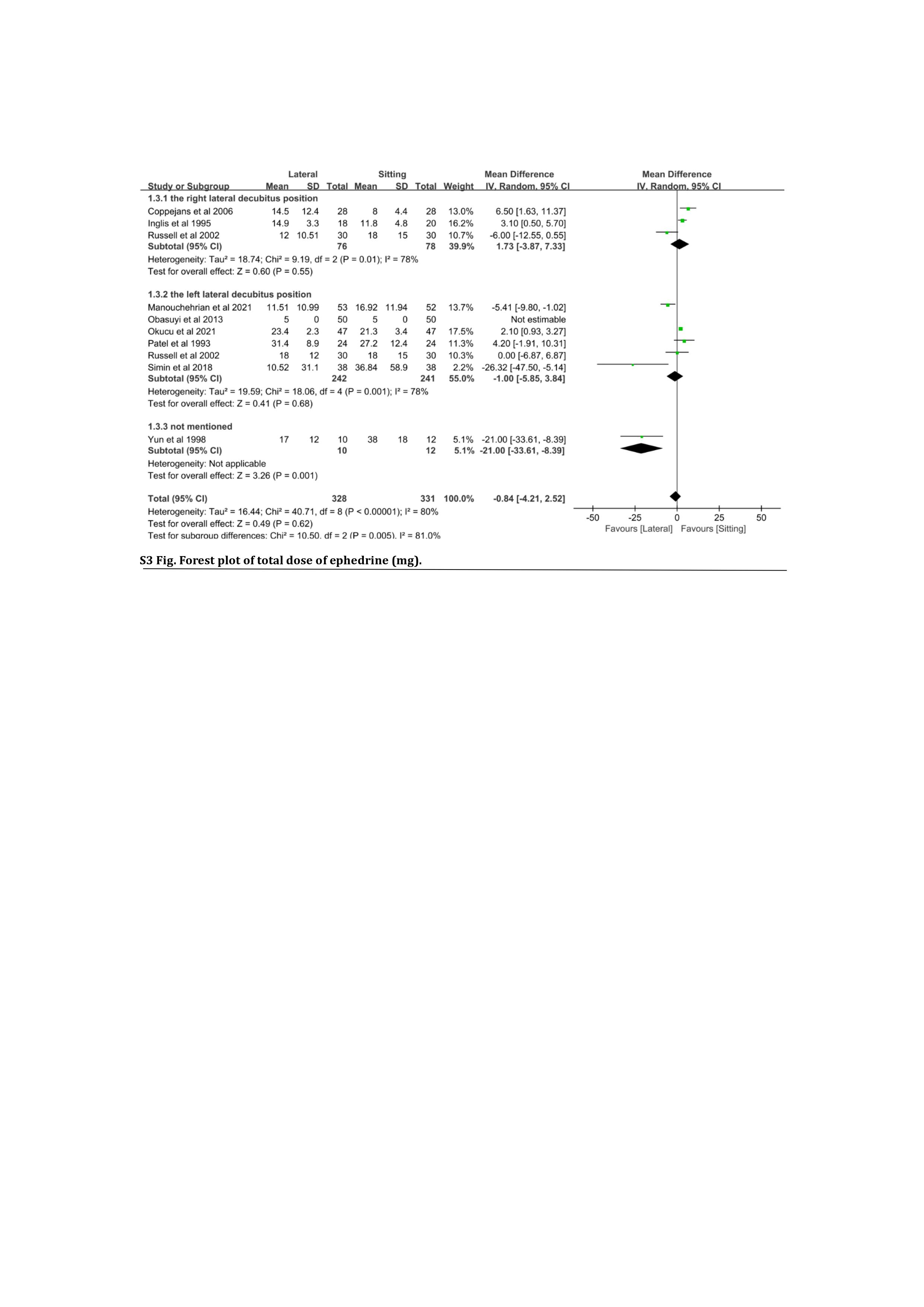

Supplement: Supplementary file 3 [file Image_3.JPEG]

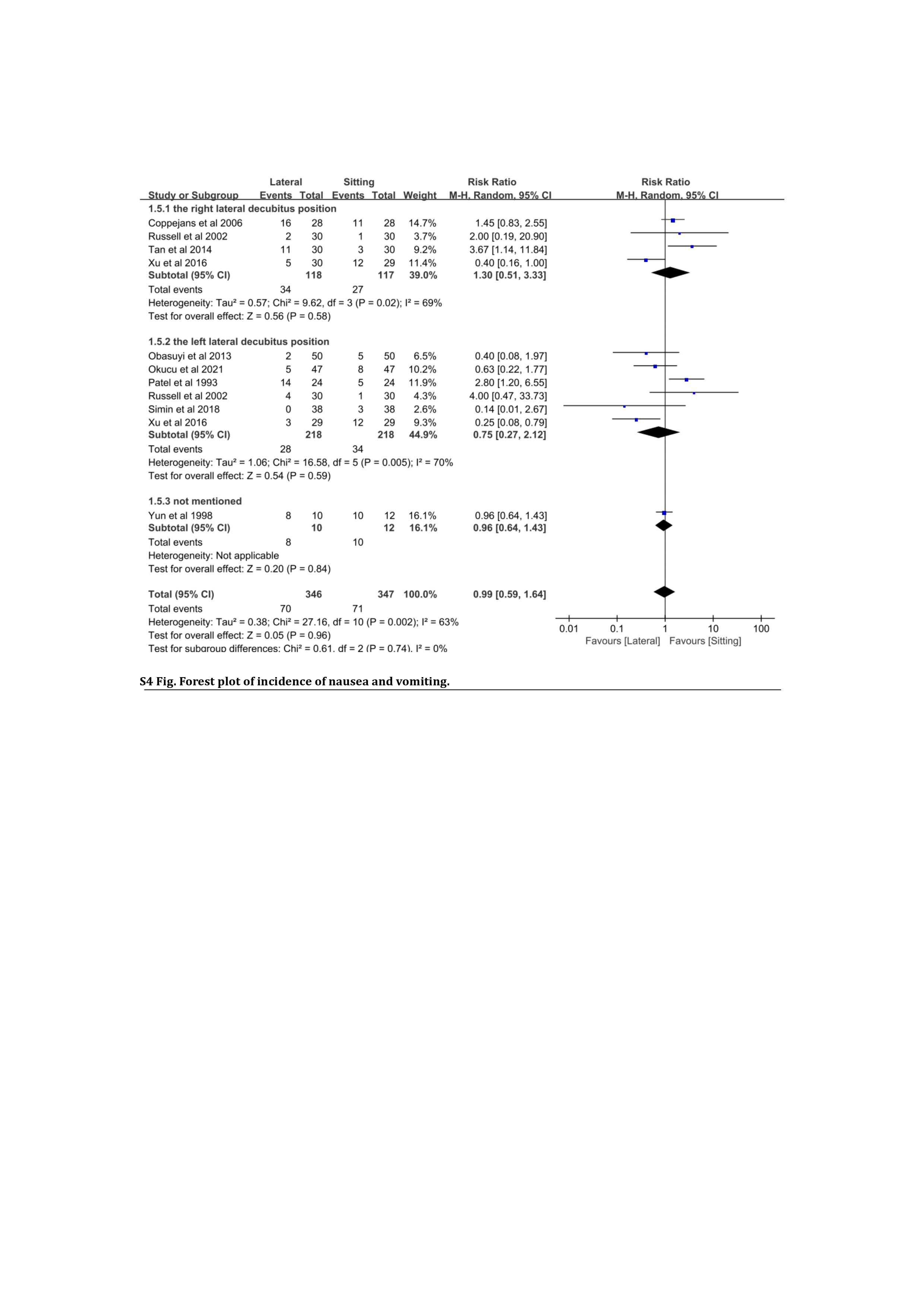

Supplement: Supplementary file 4 [file Image_4.JPEG]
